# Rituximab as a front-line therapy for adult-onset minimal change disease with nephrotic syndrome

**DOI:** 10.18632/oncotarget.25612

**Published:** 2018-06-22

**Authors:** Roberta Fenoglio, Savino Sciascia, Giulietta Beltrame, Paola Mesiano, Michela Ferro, Giacomo Quattrocchio, Elisa Menegatti, Dario Roccatello

**Affiliations:** ^1^ Nephrology and Dialysis Unit, S. Giovanni Bosco Hospital and University of Turin, Turin, Italy; ^2^ Department of Clinical and Biological Sciences, Center of Research of Immunopathology and Rare Diseases, Coordinating Center of the Network for Rare Diseases of Piedmont and Aosta Valley, Department of Clinical and Biological Sciences, University of Turin, Turin, Italy

**Keywords:** adult minimal change, rituximab, nephrotic syndrome, CD20, monoclonal antibody, Immunology

## Abstract

Minimal change disease (MCD) accounts for 15% of adult nephrotic syndrome (NS) cases. Adult-MCD patients may have more severe clinical features than pediatric patients. In children, Rituximab (RTX) has been used since 2006 to treat frequently relapsing NS. In adults, data about the efficacy of RTX for MCD are limited. We report our experience on the use of RTX in adult biopsy-proven MCD. Our series includes 6 adult patients (2 males and 4 females), age 45–73 years, treated with RTX (4 weekly doses of 375 mg/m^2^). Proteinuria decreased from 11,2 (23–4.8) g/24 hours to 0.6 (0–2) g/24 hours after 6 months, and to 0.4 (0–1, 4) g/24 h in the 4 pts with the longer follow-up. Creatinine decreased from 1.95 (0.5–5) mg/dl to 0.88 (0.6–1.3) mg/l. Five patients achieved a complete renal remission, while in 1 pt proteinuria decreased by 75%. RTX successfully depleted CD19 lymphocytes in 100% of pts for at least 6 months. No clinically relevant adverse events have been observed. This case series shows a remarkable efficacy of RTX in treatment of MCD. RTX can be an attractive alternative both in recurrent forms and in induction-therapy of MCD. RTX may be preferentially used in patients at a high risk of development of the adverse effects of corticosteroids and should be considered as an alternative option in patients with recurrent NS. Additional data are needed to inform clinical practice on how best to use RTX in this patient population, so that definitive randomized trials can be planned.

## INTRODUCTION

Minimal change disease accounts for 15% of adult cases of nephrotic syndrome [1].

Because of its high prevalence in children, most studies on the natural course, treatment, and prognosis of MCD have focused on pediatric population. Although the exact etiology of MCD remains unknown, dysregulation of the immune system is thought to be an important factor in pathogenesis [2]. In general, MCD is considered a self-limiting and relatively benign disease, and the risk of end-stage renal disease (ESRD) is extremely low. On the other hand, adult-onset MCD patients (pts), especially those who are found to have focal segmental glomerulosclerosis (FSGS) on a second kidney biopsy, might experience a progression to ESRD [3]. Adult-onset MCD patients may have more severe clinical features than pediatric MCD pts. Remission induction is of great relevance to these patients. Significant practice variability exists in the management of adult MCD [4]. Several therapeutic approaches have been attempted in nephrotic adult-MCD, but the best treatment, able to assure rapiddly induced, long time remission with few adverse events, is far from being established. There are only few randomized clinical trials (RCTs) addressing therapy for these conditions in adults. Steroids are the mainstay of treatment. Glucocorticosteroids have been recommended as a first-line therapy by the Kidney Disease Improving Global Outcomes (KDIGO) guidelines [5]. Adult-onset MCD patients have been reported to be at higher risk of acute kidney injury with delayed response to glucocorticosteroids as compared to pediatric pts [6]. Only 30% of adult pts achieve remission over the 8 week course of therapy which is thought to be sufficient in the majority of pediatric cases. Furthermore, among adults who do respond to corticosteroid therapy, 25% will experience a frequent relapsing course and 30% become steroid-dependent [7]. Repeated and prolonged steroid therapy is associated to a number of side effects [8].

A variety of approaches to the management of MCD have been attempted. Immunosuppressive (IS) agents, such as calcineurin-inhibitors [9], cyclophosphamide [10] and mycophenolate mofetil [11], have been used. Cyclosporin-A is usually effective, but relapses often occur after therapy discontinuation. Moreover, the administration of calcineurin-inhibitors is frequently associated with renal dysfunction, hypertension, hyperlipidemia and gingival hyperplasia [12, 13]. Mycophenolate-mofetil may reduce the frequency of relapses, but its use can be limited by gastrointestinal adverse effects. Cyclophosphamide may induce long term remission of MCD in about 50% of patients, but cytotoxicity and infertility limit its use.

Rituximab (RTX) has been employed in children since 2006 in order to treat frequently relapsing NS [14]. RTX is currently approved for the treatment of CD20-positive lymphoma, rheumatoid arthritis and ANCA-associated vasculitides [15]. It is increasingly being used off-label in a variety of autoimmune and renal disorders [16] such as membranous nephropathy [17, 18] and IgA vasculitis [19]. While data regarding the efficacy of RTX in adult MCD are limited, it seems to be effective in reducing frequency of relapses and concomitant immunosuppression in 65–85% of patients [20]. However, different dosage and regimen (single flat dose of 500; 1,000 mg at 1 or 2 time-points, or 375 mg/m^2^ once weekly for 4 weeks), mixed populations (steroid-dependent and/or steroid-resistant patient samples) and diverse scheme (induction or maintenance) make RTX effects difficult to interpret. Whether RTX is best used to induce or maintain remission and in which pts, whether repeated doses improve response rate, and which protocol should be used remain to be established.

Herein we describe the effects of RTX given alone as a front induction therapy in a series of adult biopsy-proven MCD patients.

## RESULTS

**Figure 1:**
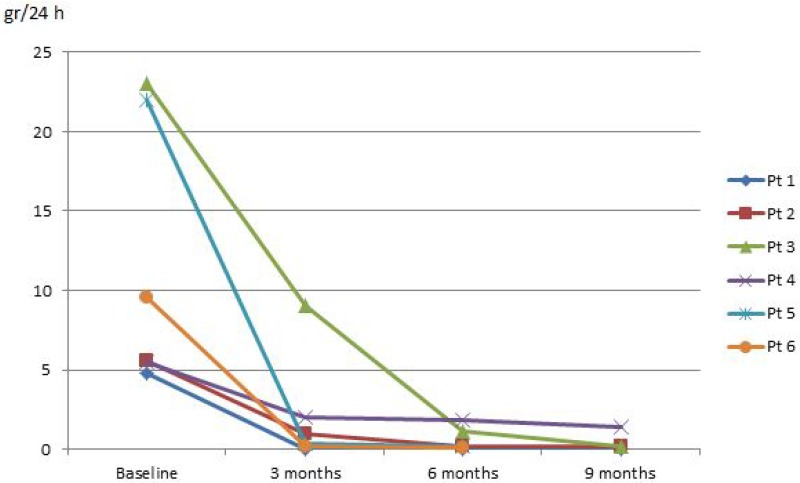
Evolution of proteinuria

Mean age of the patients was 62.7 years (min 45–max 73) (Table 1). All pts were treated with RTX as first-line therapy. Three pts reached a complete remission within 3 months, 1 pt within 6 month, and 1 pt within 9 months. In these pts, the complete remission persisted during the entire follow-up period. In the remaining pt (pt # 4) the decrease in urinary protein excretion was >75% after 9 months (Figure 1) and remained unchanged after 1 year.

**Table 1:** Demographic and clinical baseline characteristics of patients

	Pt 1	Pt 2	Pt 3	Pt 4	Pt 5	Pt 6
Sex	F	M	M	F	F	F
Age at diagnosis (years)	45	59	72	61	73	66
Duration of follow-up (months)	36	34	30	12	8	9
Urinary protein (g/day)	4.8	5.6	23	5.5	22	9,6
Albumin (g/dL)	2.1	2.3	1.3	2.9	1.7	2
Total cholesterol (mg/dL)	347		325	416	468	208
Creatinine (mg/dL)	0.8	1.6	3.2	0.5	5	0.6
White blood cell count (u/mm^3^)	4.280	8.280	6.210	8.930	9420	7190
Lymphocytes (×1000/ul)	1070	1010	1480	1420	603	2380
CD19 (/mm^3^)	77 (7.19%)	42 (4.15%)	148 (10%)	258 (12.8%)	96 (16%)	229 (8.3%)
CD20 (/mm^3^)	77 (7.17%)	42 (4.15%)	145 (9.84%)	255 (12.7%)	96 (16%)	229 (8.3%)
IgG (mg/dL)	373	794	229	1414	184	537
IgA (mg/dL)	124	137	181	130	152	146
IgM (mg/dL)	221	42	99	37	298	165

A significant increase in serum albumin and IgG levels were found during follow-up as compared to the baseline values (Table 2). Three pts had an impaired renal function at the diagnosis. In 2 of them, creatinine values normalized within 1 month after RTX administration and in the 3th pt (#5) within 3 months. None of these patients relapsed during the follow-up of 8–36 months.

**Table 2:** Laboratory data at last follow-up

	Pt 1	Pt 2	Pt 3	Pt 4	Pt 5	Pt 6
Urinary protein (g/day)	0	0.1	0.1	1.3	0.15	0
Albumin (g/dL)	4.2	3.9	4.5	3.2	4.3	3.6
Creatinine (mg/dL)	0.8	0.9	0.8	0.6	1.2	0.6
White blood cell count (u/mm^3^)	4.360	7740	9700	12330	5500	7190
Lymphocytes (×1000/ul)	1170	1160	2910	1190	2250	2380
IgG (mg/dL)	778	1079	987	1378	740	840
IgA (mg/dL)	148	137	178	147	152	140
IgM (mg/dL)	110	45	70	34	248	145

With regards to the safety, none of the 6 pts experienced infusion-related or hematological reactions, nor developed infections by the end of follow-up.

## DISCUSSION

Many therapeutic approaches for adult MCD have been attempted, but the ideal regimen is far from being established. In contrast to the known involvement of T cells in minimal-change nephrotic syndrome (MCNS), the role of B-lymphocytes is not defined yet [21]. B-cell biology, however, has attained attention since RTX, a monoclonal antibody directed to CD20-bearing cells, has shown some therapeutic response in the treatment of MCD. In recent years, it has been reported that B-cells also have regulatory functions. The regulatory B-cell stimulates both effector and regulatory T-cells [22]. In other auto-immune diseases, regulatory T-cell function improves after treatment with RTX [18, 23]. The mechanism of action is unknown, B-cell depletion may lead to a new balance between the T-cell subsets. This balance can be established even after repopulation of the peripheral B-cells. A possible mechanism is that T-cells that interact with B-cells are removed simultaneously after treatment with RTX [24, 25].

Previous findings have shown that CD23 increases in patients with nephrotic syndrome [26]. Recent studies have reported that increased Interleukin-13, a cytokine associated with type 2 T-helper cells, can lead to podocyte injury and may induce a MCNS-like phenotype [27]. B-cells are essential in CD4 T-cell activation for antigen presenting and providing co-stimulation signals. Tokunaga *et al*. [28] showed that RTX decreases CD40- and CD80-expression in activated B-cells in patients with systemic lupus-erythematosus, and down-regulates CD40L and CD69 on CD4-positive cells. Successful induction of remission following RTX treatment in some cases of MCD provides evidence that the interference of B- and T-cells is a potential strategy for MCD treatment [29]. On the other hand, it is also possible that the B-cells play a direct role in the pathogenesis of MCD, but evidence of this hypothesis is lacking. Moreover, RTX directly affects T-cells, affecting naïve/memory balance [30]. Beside immunological explanation, RTX may exert a direct effect in the kidney. A recent study shows that RTX may stabilize the cytoskeleton of the podocyte [31].

To our knowledge this is the first report of adult minimal change disease treated with RTX as first-line therapy without the association of steroid/immunosuppressive drugs. All patients met the clinical criteria of nephrotic syndrome although in some of them proteinuria values were not so high as commonly expected. Three patients had a renal failure. The minimal change disease was biopsy proven in all patients. In our cohort, one patient had a partial remission and 5 patients had complete response within 6 months, and remained in remission at the end of follow-up after a single course of anti-CD20 therapy (median follow-up 21.5 months, 8–36 months). We cannot exclude by certainty that the patients could experience a spontaneous remission. Actually, every patient did respond to the RTX regimen with a time to remission consonant with the effectiveness timing of the drug.

An unanswered question is whether RTX treatment should be repeated after repopulation of the peripheral B-cell population in the effort to achieve protracted remission. Published studies vary with regard to the dose and number of doses of RTX that are needed to achieve long-term remission of MCD. A single dose of RTX achieved a good response in the series of 12 patients described by Kamei, but the relapse rate was 75% [32]. Another study found that the time to NS relapse was longer when more than three doses were given at the beginning [33].

In our series, 5 patients experienced a sustained remission over a prolonged follow-up and did not required repeated infusions. They remained relapse-free despite the recovery of B-cell count, suggesting that, as in other immunologic disorders [18, 26], RTX restores patients' immunologic balance. This data differs from previous experiences reported in literature. Protracted remission could be due to the greater dose regimen (4 doses of 375 mg/m2) we used compared to other published series.

Other possible explanations include the characteristics of our patient sample which differ from previous studies, i.e., older age and naive cases compared to steroid-dependent and frequent relapsing forms.

Further studies are needed to confirm this hypothesis.

## MATERIALS AND METHODS

We describe the effects of the treatment with RTX in a cohort of six adult patients with biopsy-proven MCD. The kidney histology was normal and the immunofluorescence negative; the electron microscopy was not done in all patients. Secondary causes were carefully ruled out. All patients received RTX as first line-therapy, without the association of corticosteroids or any other immunosuppressive agents. All the patients were treatment naive. They were treated with 4 doses of 375 mg/m^2^ RTX with a 1-week interval. All patients had definite contraindications to steroid therapy (diabetes, BMI > 30, psychosis). All patients had a nephrotic syndrome. Renal function was normal in 3 pts, while the other 3 had renal impairment. The pressure values were normal and none of them was tacking RAS blockade. The clinical characteristics of the patients at baseline and at last follow-up are summarized in Tables 1 and 2. Laboratory parameters, including hepatitis-C and hepatitis B markers (hepatitis-B surface antigen and antibody, and hepatitis-B core antibody) were detected in all patients. Two pts who were Quantiferon-TB positive, despite the absence of other signs of disease. They were treated with isoniazid for 6 months.

## CONCLUSIONS

The major limitations of the study include its retrospective nature and the small sample. Nevertheless, our results support the encouraging effects reported in a pediatric setting. We suggest that RTX, given alone, is an effective and safe front-line therapy in adult patients with MCD. RTX might be preferred in patients at high risk of developing the several adverse effects of corticosteroids. Our study sets the scene for future large-scale prospective studies to further investigate the use of RTX in the context of the management of adult onset of minimal change disease.

## Abbreviations

MCD: minimal change disease
NS: nephrotic syndrome
ESRD: end-stage renal disease
PTS: patients
FSGS: focal segmental glomerulosclerosis
RTX: rituximab
MCNS: minimal-change nephrotic syndrome (MCNS)
